# A Multi-causal Model for Chronic Malnutrition and Anemia in a Population of Rural Coastal Children in Ecuador

**DOI:** 10.1007/s10995-019-02837-x

**Published:** 2019-12-14

**Authors:** María F. Rivadeneira, Ana L. Moncayo, Betzabé Tello, Ana L. Torres, Gladys J. Buitrón, Fabricio Astudillo, Todd R. Fredricks, Mario J. Grijalva

**Affiliations:** 1 Instituto de Salud Pública, Facultad de Medicina, Pontificia Universidad Católica del Ecuador, Apartado, 1701-2184 Quito, Ecuador; 2 Centro de Investigación para la Salud en América Latina (CISeAL), Escuela de Ciencias Biológicas, Facultad de Ciencias Exactas y Naturales, Pontificia Universidad Católica del Ecuador, Apartado, 1701-2184 Quito, Ecuador; 3 Facultad de Medicina, Pontificia Universidad Católica del Ecuador, Apartado, 1701-2184 Quito, Ecuador; 4 Escuela de Ciencias Físicas y Matemática, Facultad de Ciencias Exactas y Naturales, Pontificia Universidad Católica del Ecuador, Apartado, 1701-2184 Quito, Ecuador; 5 Escuela de Geografía, Facultad de Ciencias Humanas, Pontificia Universidad Católica del Ecuador, Apartado, 1701-2184 Quito, Ecuador; 6 Department of Family Medicine, Heritage College of Osteopathic Medicine, Ohio University, Athens, OH 45701 USA; 7 Infectious and Tropical Disease Institute, Department of Biomedical Sciences, Heritage College of Osteopathic Medicine, Ohio University, Athens, OH 45701 USA

**Keywords:** Malnutrition, Children, Determinants, Rural communities, Ecuador

## Abstract

**Objectives:**

Chronic malnutrition and anemia are prevalent in developing countries. This research aimed to determine the prevalence of chronic malnutrition and anemia and their associated factors in children under five using a multi-causal model in a rural community in the coast of Ecuador.

**Methods:**

The study included 314 children under 5 years old who were residents of San Isidro, Ecuador. Indicators of chronic malnutrition and anemia were identified. Mothers/caregivers were surveyed on socio-economic and environmental conditions, feeding and care practices, access to health services and biological characteristics. Bivariate and multivariable Poisson regression were performed.

**Results:**

The prevalence was 12.42% (n = 39) for chronic malnutrition and 16.98% (n = 54) for anemia. There was a significant and independent association between chronic malnutrition and family income less than $80 USD per month (Prevalence Ratio [PR] = 2.74, 95% CI 1.04, 7.20), maternal height less than 150 cm (PR 3.00, 95% CI 1.69, 5.32) and residence in a household with more than 4 children (PR 3.05, 95% CI 1.48, 6.29). Anemia was 2.57 times higher (95% CI 1.17, 5.65) in children with more than two episodes of diarrhea in the last 6 months. Prenatal care (5 to 8 visits) provided a protective effect for anemia (PR 0.48, 95% CI 0.27, 0.89).

**Conclusions for Practice:**

Findings support the need for comprehensive interventions targeted toward chronic malnutrition and anemia in children from rural coastal communities. Improvement of socioeconomic conditions, family planning, prenatal care and reduction of diarrheal diseases should be prioritized.

## Significance

*What is already known on this subject?* Ecuador is one of Latin America countries with the highest rates of chronic malnutrition and anemia in children under 5 years, especially in rural areas. *What this study adds?* This study used a multi-causal model associated with chronic malnutrition and anemia in rural coastal children population and implemented a multivariate regression modeling taking in account the hierarchical relationships between the potential determinants of malnutrition. This will help to design comprehensive interventions targeted to decrease malnutrition and anemia in children living in rural coastal communities.

## Introduction

Chronic malnutrition and childhood anemia still remain significant public health problems worldwide (Aheto et al. 2015; Black et al. 2013; Fikadu et al. 2014). Globally, it was estimated that 155 million children under 5 years of age (22.9%) experienced a delay in growth in 2016 (UNICEF et al. 2017). The worldwide prevalent cases of iron-deficiency anemia in children and adolescents 19 years or younger was 713 million (Kassebaum et al. 2017). In 2012, Ecuador had one of the highest rates of chronic malnutrition (25.3%) and anemia (25.7%) in children under 5 years in Latin America (Freire et al. 2014).

Malnutrition in childhood has many adverse consequences for child survival and long-term well-being. Child malnutrition is associated with increased infections, poor psychomotor development, poor school performance, increased risk of chronic disease in adulthood, lower capacity for work and lower quality of life (Kassebaum et al. 2017). Iron-deficiency anemia is one of the most important causes of years lived with disability (YLDs) in children and adolescents (Kassebaum et al. 2017).

Pediatric malnutrition and anemia are multifactorial. The multi-causal model of infant malnutrition established by the United Nations Children’s Fund (UNICEF) is comprised of immediate, underlying and basic determinants (UNICEF 1990). The immediate determinants include inadequate dietary intake and the presence of infectious diseases (Black et al. 2013; Fikadu et al. 2014). The underlying causes are food insecurity; inadequate care and feeding practices for children; inadequate health care and unhealthy household and surrounding environments (Aheto et al. 2015). The basic causes include structural factors such as household access to adequate quantity and quality of resources (land, education, employment, income) and sociocultural, economic and political context (Tariku et al. 2017).

We conducted a survey in San Isidro parish, a rural community in the coastal province of Manabí, Ecuador. The aim was to determine the prevalence of chronic malnutrition and anemia and associated factors in children under five using a multi-causal model. The result of this study could help in the design of strategies to reduce malnutrition in this pediatric rural population.

## Methods

### Study Area

We conducted a cross-sectional study in September 2017 in San Isidro, a rural parish of Manabí Province, located on the central coast of Ecuador, at 140 m above sea level (GAD San Isidro 2014). San Isidro has a total area of 296 km^2^ and 11,411 inhabitants making up 2738 households. The annual average temperature is about 26 °C (GAD San Isidro 2014). In 2010, the Unmet Basic Needs (UBN) poverty rate per person was 86.5% (INEC 2010). San Isidro has an agricultural economy centered on the cultivation of corn, fruits, cocoa, coffee, bananas, rice, and cotton (GAD San Isidro 2014).

### Study Population and Sample Size

The sample size consisted of 314 children aged 0–59 months. This sample size was determined by assuming a 24.7% prevalence rate of chronic malnutrition in rural coastal children of Ecuador (Freire et al. 2014) with a confidence level of 95%, a precision of 5% and 10% for nonresponse and missing data. Children were recruited at day care centers and schools. Children who received treatment for infectious diseases or who were hospitalized in the 2 weeks prior to the survey were excluded from the study. Children with birth complications such as prematurity, congenital defects or another condition that impairs growth and development were also excluded.

### Ethics

After receiving a description of the study, written informed consent was obtained from mothers or guardians of the children. Protocol was approved by the Institutional Review Board (IRB) from the Pontifical Catholic University of Ecuador (protocol number CEISH-297-2017) and from the Ministry of Public Health of Ecuador (Protocol Number MSPCURI000216-3-etapa 1).

### Procedures

The survey used in this study was based on the Spanish version of the Questionnaire for children under five from the Multiple Indicator Cluster Survey (MICS) designed by UNICEF (UNICEF 2018) and Encuesta Nacional de Salud y Nutrición (ENSANUT) (Freire et al. 2014). The survey was validated in San Isidro to ensure cultural appropriateness and was administrated to obtain data about demographic, socio-economic, environmental, and biological characteristics; feeding and childcare practices; and use of health services. Data were collected by trained nutrition students in face-to-face interviews of the surveyed children’s primary caregivers.

Children and mothers were weighed on portable electronic microscales (ADE, model M320600, Hamburg, Germany) and height was measured using a portable stadiometer (SECA model SECA 213, Hamburg, Germany). Recumbent infant length was measured with a length board (ADE model MZ10027-1, Hamburg, Germany). Variations of 100 g were permitted for weight and 0.1 cm for length/height. The mean of two measurements was considered as the final measurement. The instruments were calibrated periodically. Researchers followed the recommended technical standards and criteria throughout all steps of the anthropometric evaluation (Freire et al. 2014). Z-scores for length/height-for-age (LAZ), weight-for-age (WAZ) and weight-for-length/height (WHZ) were calculated using the WHO 2006 growth standard references (de Onis et al. 2007). Venous blood (3 ml) was drawn from each child and hemoglobin values were determined with a spectrophotometer.

### Analysis Model and Variable Description

The dependent variable constituted stunting (LAZ <  − 2 SD), as a proxy of chronic malnutrition and a categorical variable was constructed (yes/no). Anemia was defined as a haemoglobin level in blood of < 110 g/l for children 6–59 months of age at sea level (WHO 2011).

**Fig. 1:**
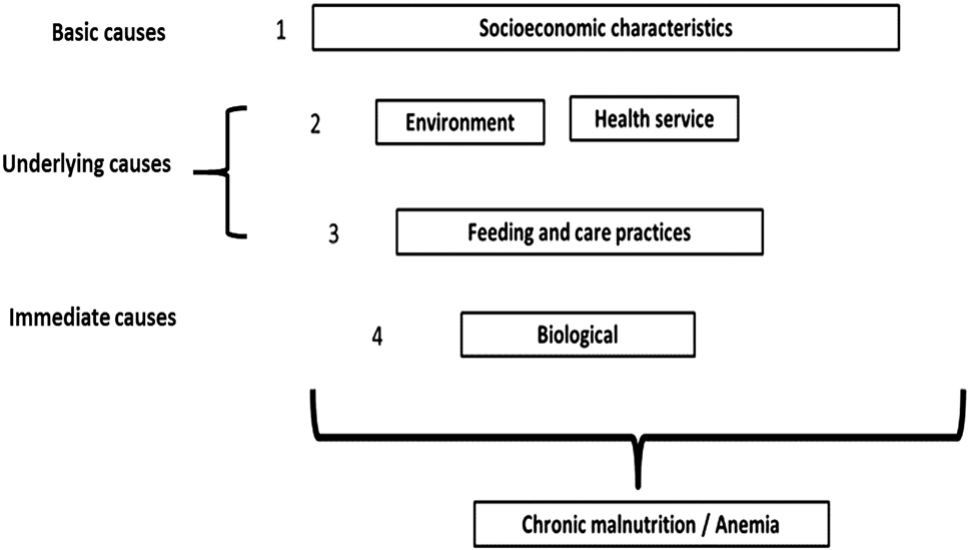
Model of hierarchical analysis according to determinants associated with chronic malnutrition and anemia in children

Analysis follows a multi-causal model (UNICEF 1990) that identifies basic, underlying, and immediate causes of chronic malnutrition (Fig. 1). Basic causes can result in lack of income, education and knowledge. Underlying causes are related to the impossibility of fulfilling unmet basic needs and immediate causes have to do with survival conditions and biological characteristics.

From this model, the independent variables were classified into four blocks or levels of analysis: Block 1 which includes the basic causes: the socioeconomic variables (education of mother and father, number of dependents in the home and family income in United States dollars); Block 2 and 3 which include the underlying causes. Block 2 includes the environmental characteristics (overcrowding, water supply, excreta disposal, source of water used for drinking) and variables related to health services (timing of initial prenatal care visit, number of prenatal care visits, post-partum care, medical monitoring of the child after birth and number of well-baby checkups; Block 3 includes feeding and care practices (exclusive breastfeeding in the first 6 months from birth, continuous breastfeeding after 6 months, age which food was introduced, food diversity or consumption of at least four food groups 1 day prior to the survey for children older than 6 months; practices of care included treatment of water for human consumption, whether the surveyed child had received any treatment in the last episode of diarrhea or of any other disease; Block 4, the immediate causes, which includes the biological characteristics (mother’s height under 150 cm, child height at birth less than 45 cm, the number of children by mother, the presence of an intergenesic period related to the child being surveyed, the presence of diarrheal episodes in the last month and in the last 6 months, episodes of respiratory infections during the last 6 months, the number of hospitalizations in the last year, and the number of episodes of parasitic infections in the last year (Fig. 1).

### Statistical Analysis

The OpenClinica software was used in data entry and STATA version 14.0 (StataCorp LLC, Texas, USA) was used in the analysis. Descriptive statistics were used to summarize the anthropometric Z-scores according to the age and gender and to determine the prevalence of chronic malnutrition and anemia in the study population. Bivariate analysis was performed to determine associations between outcomes and independent variables. Further analysis was done using multivariable Poisson regression (PR and 95% CI) taking in account the hierarchical relationships between the potential determinants of malnutrition (Victora et al. 1997). According to the conceptual model, the analysis started at block 1 (basic determinants), and the variables that were significant (p < 0.20) were maintained for the subsequent model in which all underlying determinants (Block 2 and 3) were added. Likewise, variables that were significant (p < 0.1) were maintained for the next model including all variables of immediate determinants (Block 4). Finally, only significant variables (p < 0.05) were maintained for the final model. The variables of Block 1 (socioeconomic) were maintained as adjustment variables for the rest of the model.

## Results

### Sample Characteristics

In this survey, 343 children 0–59 months of age were recruited of which 10 (2.9%) were excluded from the study because they did not meet the inclusion criteria. Of those 333 children, 314 (94.3%) had complete data for chronic malnutrition and anemia. Characteristics of the sample are shown in Table 1. The study included 144 (45.9%) males and 170 (54.1%) females with a mean age of 39 months (range 2–59 months). The average age of the mothers was 28 years (range 15–54). Approximately half of the families (54.6%) had monthly incomes between $80 and $300 USD and 20% had income below $80. The mothers had higher levels of education than the fathers (university/high school: 57.4% vs. 39.2%). The primary water supply identified was river or watershed sources (52%). Latrine or septic tanks were the most common method of excreta disposal (78.9%). A low percentage of mothers (13%) had less than five prenatal check-ups. Seventy-nine percent of children 6 months of age or older had received exclusive breastfeeding, but only 49% of children over age of one continued to be breastfed. Twenty-two percent of children were introduced to solid food before 6 months of age. The percentage of children that had more than two episodes of diarrhea and respiratory infections in the 6 months preceding the survey was 20.4% and 51%, respectively.

**Table 1:** Bivariate analysis of the association between health determinants and chronic malnutrition and anemia in children under 5 years of age, San Isidro, Ecuador, 2017

	Sample	Chronic malnutrition	Anemia
	N (%)	PR (95% CI)^a^	PR (95% CI)^a^
Socioeconomic characteristics			
Family income			
> $300	76 (25.2)	Reference	Reference
$80.1–$300	165 (54.6)	1.92 (0.75, 4.92)*	1.37 (0.64, 2.91)
< $80	61 (20.2)	3.13 (1.18, 8.32)**	3.09 (1.46, 6.56)**
Maternal education status			
University	32 (10.7)	Reference	Reference
High school	140 (46.7)	4.73 (0.66, 34.01)*	4.69 (0.66, 33.54)
Primary school/illiterate	128 (42.7)	4.09 (0.56, 29.71)*	6.54 (0.92, 46.29)*
Paternal education status			
University/high school	119 (39.2)	Reference	Reference
Primary school	146 (49.2)	1.65 (0.84, 3.25)*	1.07 (0.61, 1.89)
Illiterate	32 (10.8)	1.59 (0.59, 4.27)	2.30 (1.18, 4.46)**
Number of dependents			
1–4 people	157 (52.2)	Reference	Reference
5–6 people	90 (29.9)	2.47 (1.24, 4.90)**	0.71 (0.38, 1.29)
> 6 people	54 (17.9)	2.19 (0.98, 4.93)*	0.96 (0.50, 1.84)
Environmental characteristics			
Overcrowding			
No	98 (35.3)	Reference	Reference
Yes	180 (64.7)	0.78 (0.40, 1.52)	2.05 (1.02, 4.09)**
Water supply			
Public supply system	119 (39.9)	Reference	Reference
Pipeline	17 (5.7)	0.64 (0.09, 4.67)	2.31 (0.62, 7.73)
Distributor car	7 (2.3)	3.12 (0.85, 11.46)*	1.87 (0.27, 12.82)
River or watershed	155 (52.0)	1.69 (0.87, 3.31)*	3.00 (1.51, 5.97)**
Excreta disposal			
Public sewer	53 (17.7)	Reference	Reference
Latrine or septic tank	236 (78.9)	2.47 (0.78, 7.76)*	1.91 (0.79, 4.60)*
Outdoors	10 (3.3)	4.91 (1.13, 21.25)**	2.70 (0.74, 9.80)*
Source of water used for drinking			
Water bottle	127 (42.1)	Reference	Reference
Public supply system	72 (23.8)	0.83 (0.33, 2.10)	0.61 (0.25,1.48)
River	66 (21.9)	2.17 (1.10, 4.31)**	1.78 (0.97, 3.36)*
Other	37 (12.2)	1.32 (0.50, 3.46)	2.38 (1.25, 4.53)**
Health services related variables			
First prenatal care (trimester of pregnancy)			
First	256 (87.9)	Reference	Reference
Second or third	35 (12.1)	2.07 (1.02, 4.09)**	1.75 (0.96, 3.17)*
Number of prenatal care visits			
< 5	36 (12.6)	Reference	Reference
5–8	153 (53.7)	0.49 (0.23, 1.07)*	0.36 (0.20, 0.66)**
9–18	96 (33.7)	0.57 (0.26, 1.30)*	0.54 (0.30, 0.99)**
Postpartum care			
Yes	232 (80.0)	Reference	Reference
No	58 (20.0)	0.79 (0.34, 1.81)	0.88 (0.47, 1.66)
Medical monitoring of the child after birth			
Yes	284 (94.7)	Reference	Reference
No	16 (5.3)	0.49 (0.07, 3.37)	0.76 (0.20, 2.82)
Number of well-baby checkups			
> 2	148 (49.3)	Reference	Reference
1–2	64 (21.3)	1.50 (0.77, 2.90)	0.66 (0.35, 1.26)
None	88 (29.3)	0.64 (0.28, 1.27)	0.39 (0.19, 0.81)*
Feeding and care practices			
Exclusive breastfeeding			
Yes	231 (78.9)	Reference	Reference
No	65 (22.0)	0.76 (0.35, 1.64)	1.16 (0.66, 2.06)
Continuous breastfeeding			
Yes	131 (48.7)	Reference	Reference
No	138 (51.3)	0.63 (0.33, 1.19)	0.65 (0.33, 1.27)
Initiation of food introduction (age) (months)			
< 6	65 (22.0)	Reference	Reference
6	133(44.9)	1.38 (0.61, 3.12)	0.81 (0.43, 1.53)
> 6	98 (33.1)	1.25 (0.53, 2.98)	0.92 (0.48, 1.76)
Food diversity			
Yes	251 (86.0)	Reference	Reference
No	41 (14.0)	0.33 (0.08, 1.32)	2.08 (1.20, 3.58)**
Treatment of water for consumption			
Boil	146 (49.2)	Reference	Reference
Filter	25 (8.4)	0.87 (0.28, 2.70)	1.22 (0.60, 2.49)
Chlorine	27 (9.1)	0.54 (0.13, 2.16)	0.87 (0.37, 2.05)
Nothing	99 (33.3)	0.94 (0.49, 1.79)	0.44 (0.22, 0.88)**
Received any treatment last episode of diarrhea			
Yes	134 (87.0)	Reference	Reference
No	20 (13.0)	2.51 (0.35, 17.89)	0.81 (0.35, 1.85)
Received any treatment last episode other disease			
Yes	196 (80.7)	Reference	Reference
No	47 (19.3)	0.89 (0.39, 2.05)	1.15 (0.54, 2.43)
Biologic characteristics			
Maternal height < 150 cm			
No	207 (70.9)	Reference	Reference
Yes	85 (29.1)	3.04 (1.69, 5.49)***	1.14 (0.67, 1.94)
Height of the child at birth < 45 cm			
No	125 (88.7)	Reference	Reference
Yes	16 (11.3)	4.03 (1.11, 14.63)**	0.65 (0.17, 2.52)
Number of children			
1–2	191 (62.8)	Reference	Reference
3–4	93 (30.6)	1.44 (0.74, 2.83)	0.9 (0.51,1.58)
> 4	20 (6.6)	3.88 (1.91, 7.87)***	1.64 (0.77, 3.48)
Intergenesic period^b^			
> 4 years	224 (73.2)	Reference	Reference
2–4 years	53 (17.3)	0.58 (0.21, 1.59)	0.72 (0.32, 1.61)
< 2 years	29 (9.5)	1.07 (0.40, 2.81)	1.40 (0.68, 2.85)
Diarrheal episode in the last month			
No	184 (60.3)	Reference	Reference
Yes	121 (39.7)	0.96 (0.52, 1.76)	1.50 (0.92, 2.43)*
Diarrheal episodes in the last 6 months			
None	112 (36.8)	Reference	Reference
1–2	130 (42.8)	0.66 (0.34, 1.26)	1.74 (0.93, 3.22)
> 2	62 (20.4)	0.61 (0.26, 1.47)	2.23 (1.13, 4.39)*
Episodes of respiratory infections in the last 6 months			
None	28 (9.3)	Reference	Reference
1–2	119 (39.7)	0.68 (0.27, 1.75)	1.08 (0.45, 2.61)
> 2	153 (51.0)	0.74 (0.30, 1.83)	0.91 (0.38, 2.19)
Number of hospitalizations in the last year			
None	269 (89.7)	Reference	Reference
≥ 1	7 (2.3)	1.26 (0.53, 3.00)	1.70 (0.86, 3.27)*
Episodes of parasitic infections in the last year			
None	86 (38.6)	Reference	Reference
1–2	119 (53.4)	0.67 (0.33, 1.35)	0.83 (0.44, 1.56)
> 2	18 (8.1)	0.35 (0.05, 2.50)	0.64 (0.16, 2.58)

^a^PR (95% CI) = Prevalence Ratio and 95% Confidence Interval

^b^Intergenesic period = interval between live births

*Significant differences (p < 0.20)

**Significant differences (p < 0.05)

***Significant differences (p < 0.01)

**Fig. 2:**
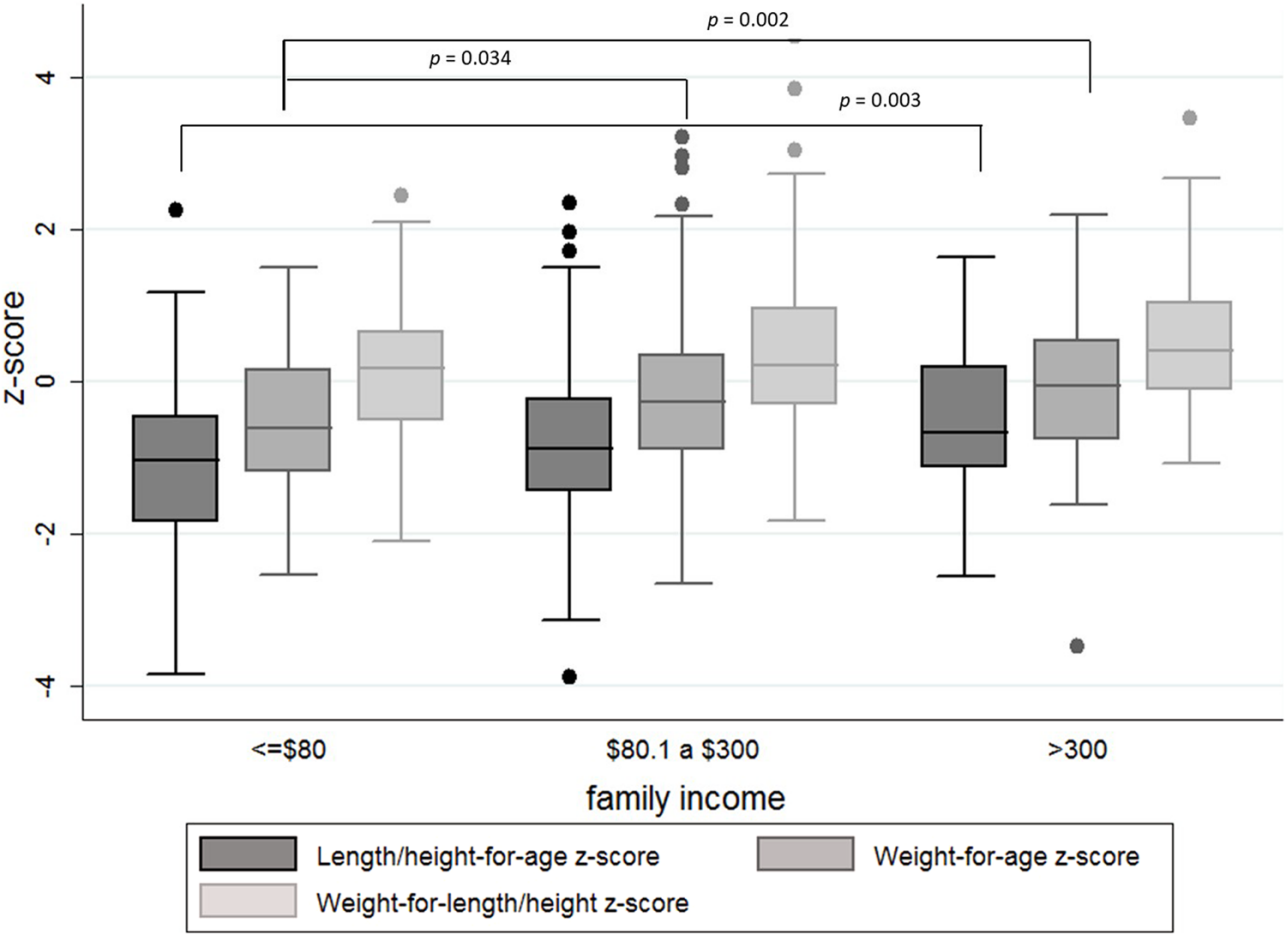
Anthropometric characteristics in children under 5 years old (z-score), stratified by family income. San Isidro, Ecuador, 2017

Table 2 shows the descriptive analysis of the anthropometric indicators and hemoglobin by sex and age. The median Z-scores were − 0.3 (interquartile range [IQR] − 0.92, 0.34) for weight-for-age, − 0.85 (IQR − 1.42, − 0.16) for height-for-age and 0.24 (IQR − 0.39, 0.93) for weight-for-height. The median for WAZ and WHZ was significantly lower (p < 0.05) for females (WAZ − 0.53, IQR − 0.97, 0.3; WHZ 0.08, IQR − 0.51, 0.76), than for males (WAZ − 0.15, IQR − 0.78, 0.48; WHZ 0.49, IQR − 0.18, 1.06). The lowest median Z-score of HAZ was observed in children between 25 and 36 months of age (HAZ − 1.16, IQR − 1.73, − 0.54). Figure 2 shows Z-scores for the anthropometric characteristics of the children by family income. The Z-scores of HAZ and WAZ were significantly lower in families with income below $80 (p = 0.01 and p = 0.006, respectively).

**Table 2:** Descriptive analysis of the anthropometric indicators and hemoglobin by age and sex, San Isidro, Ecuador, 2017

	HAZ^a^	WAZ^b^	WHZ^c^	Hemoglobin^d^
	Median (IQR)^e^	Median (IQR)^e^	Median (IQR)^e^	Median (IQR)^e^
Age (months)				
0–12	− 0.29 (− 1.05, 0.27)**	− 0.02 (− 0.55, 0.54)	0.06 (− 0.59, 0.91)	11.0 (10.0, 11.5)
13–24	− 0.85 (− 1.32, 0.29)	− 0.07 (− 0.74,0.55)	0.38 (− 0.36, 0.93)	10.9 (10.3, 11.8)
25–36	− 1.16 (− 1.73, − 0.54)	− 0.28 (− 0.96, 0.27)	0.17 (− 0.23, 1.1)	11.8 (11.3, 12.2)
37–48	− 0.89 (− 1.52, − 0.35)	− 0.51 (− 1.01, 0.23)	0.12 (− 0.41, 0.79)	11.9 (11.4, 12.4)
49–59	− 0.73 (− 1.37, − 0.18)	− 0.43 (− 0.91, 0.31)	0.27 (− 0.51, 0.89)	12.3 (11.6, 12.6)
Gender				
Male	− 0.84 (− 1.45, 0.01)	− 0.15 (− 0.78, 0.48)**	0.49 (− 0.18, 1.06)**	11.7 (11.2, 12.4)
Female	− 0.85 (− 1.38, − 0.22)	− 0.53 (− 0.97, 0.3)	0.08 (− 0.51, 0.76)	11.9 (11.3, 12.5)
Total	− 0.85 (− 1.42, − 0.16)	− 0.3 (− 0.92, 0.34)	0.24 (− 0.39, 0.93)	11.8 (11.2, 12.4)

^a^HAZ: length/height for age z-score

^b^WAZ: weight for age z-score

^c^WHZ: weight-for-length z-score

^d^Hemoglobin values in g/dl

^e^IQR: interquartile range

**Significant differences (p < 0.05)

### Chronic Malnutrition in Children Under Five and Multi-causal Model

Stunting was present in 12.4% (n = 39) of the children (51.3% in male and 48.7% in female) and the highest proportion (69.2%) was observed in children between 37 and 59 months of age.

Table 1 shows the bivariate association between independent variables and chronic malnutrition and anemia. Family income less than $80 USD per month was significantly associated with stunting in children under 5 years old (PR 3.13, 95% CI 1.18, 8.32) (Table 1). Children who live in households with no sewage system and who consume river water were 5 (95% CI 1.13, 21.25) and 2 (95% CI 1.10, 4.31) times more likely to be stunted, respectively.

The prevalence of stunting was twice as high in children whose mothers had prenatal care after the first trimester of pregnancy when compared with those children whose mothers had their first prenatal care visits in the first trimester (PR 2.07, 95% CI 1.02, 4.09). A short maternal stature, short stature of child at birth and having more than four children were significantly associated with stunting (PR 3.04, 95% CI 1.69, 5.49; PR 4.03, 95% CI 1.11, 14.63; PR 3.88, 95% CI 1.91, 7.87, respectively). After adjusting the variables using the hierarchical model, family income less than $80 (PR 2.74, 95% CI 1.04, 7.20), maternal height < 150 cm (PR 3.00, 95% CI 1.69, 5.32) and having more than 4 children (PR 3.05, 95% CI 1.48, 6.29) maintained independent and significant associations with stunting (Table 3).

**Table 3:** Multivariate regression of the association between health determinants and chronic malnutrition and anemia in children under 5 years old. San Isidro, Ecuador, 2017

	PR (95% CI)^a^
Chronic malnutrition	
Family income	
> $300	Reference
$80–$300	1.70 (0.67, 4.31)
< $80	2.74 (1.04, 7.20)**
Maternal height < 150 cm	
No	Reference
Yes	3.00 (1.69, 5.32)***
No. of children	
1–2	Reference
3–4	1.39 (0.73, 2.65)
> 4	3.05(1.48, 6.29)**
Anemia	
Number of prenatal care	
< 5	Reference
5–8	0.48 (0.27, 0.89)**
9–18	0.69 (0.36, 1.33)
Diarrheal episodes in the last 6 months	
None	Reference
1–2	1.96 (0.95, 4.02)
> 2	2.57 (1.17, 5.65)**

^a^PR (95% CI) = Prevalence Ratio and 95% Confidence Interval

**Significant differences (p < 0.05)

***Significant differences (p < 0.01)

### Anemia in Children Under Five and Multi-causal Model

Anemia was present in 16.98% (n = 54) of the study population (52.6% in female and 47.4% in male). The highest prevalence of anemia occurred in children aged 0–24 months (66.7%). In the bivariate analysis, children from families with a monthly income below $80 were more likely to have anemia than those whose families had an income above $300 (PR 3.09, 95% CI 1.46, 6.56) (Table 3). Overcrowding and use of river water for drinking increased the probability of anemia in children by two and three times, respectively (overcrowding: PR 2.05, 95% CI 1.02, 4.09 and river water: PR 3.00, 95% CI 1.51, 5.97). Five or more maternal prenatal care visits had a protective effect for anemia (PR 0.36, 95% CI 0.20, 0.66 for 5 to 8 visits, and PR 0.54, 95% CI 0.30, 0.99 for more than 8 visits). Finally, absence of dietary diversity (PR 2.08, 95% CI 1.20, 3.58) and the presence of more than two episodes of diarrhea in the 6 months prior to the survey (PR 2.23, 95% CI 1.13–4.39) were associated with a higher probability of anemia.

After multivariable analysis was performed, anemia was 2.57 times higher (95% CI 1.17, 5.65) for children with more than 2 episodes of diarrhea in the 6 months prior to the survey compared to those who did not experience any episodes (Table 3). Prenatal care (5–8 controls) provided a protective effect for anemia (PR 0.48, 95% CI 0.27, 0.89).

## Discussion

The aim of this study was to determine the prevalence of chronic malnutrition and anemia and identify their determinants using a multi-causal model in children under 5 years of age living in San Isidro. This study found a stunting prevalence of 12.42% that was associated with short stature of the mother, presence of more than 4 children and low family income. The prevalence of anemia was 16.98% and was associated with more than two episodes of diarrhea in the 6 months prior to the survey. Five or more prenatal care visits by the mother decreased the probability of anemia in her children.

Chronic malnutrition and anemia are two prevalent health problems in children from developing countries. They are the result of inadequate socioeconomic conditions, limitations in access to health care, inadequate feeding and care practices as well as related biological characteristics (Aheto et al. 2015; UNICEF 1990). The prevalence of malnutrition and anemia found in this study was lower than that registered at the national level in 2012 (25.3% and 25.7, respectively) (Freire et al. 2014). Some unique characteristics of the surveyed population could explain this result. The primary occupation in the area is crop and livestock agriculture. This could facilitate diversity and availability of food resources (INEC 2017), improving food security and therefore, the overall nutritional status of the population (Chakona and Shackleton 2017; Govender et al. 2016). In addition, it was observed that the population in San Isidro frequently access health services, both for prenatal care and for the general medical care of their children (98% of the mothers surveyed had prenatal care and 95% of the children received care after birth). In 2012, a national survey showed similar results. Only 5% of pregnant women did not have prenatal controls and this percentage increased in the rural areas (9.3%) (Freire et al. 2014). According to data of the Ministry of Public Health of Ecuador, the average number of prenatal controls in Ecuador was 4.1 in 2017 and in Manabí, the province were San Isidro is located, was 3.6 controls per pregnant women (MSP 2016). This national survey also showed that 97.7% of children in Ecuador received at least one control after birth (urban areas: 98.0% and rural areas: 97.1%) and 90.7% of children had their first control during the first month of age (Freire et al. 2014).

In this study, an optimal number of prenatal care visits (5–8) was associated with a lower prevalence of anemia in children under 5 years of age. Maternal prenatal feeding interventions and the use of micronutrients during pregnancy have a favorable effect on the nutritional status of children at birth (Svefors et al. 2018). This reinforces the importance of prenatal care as a preventive measure, and supports the need to strengthen counseling and involvement of parents in the care of their children, as a practice of prenatal control (Casale et al. 2018; Kassebaum et al. 2017; Muhoozi et al. 2018). Post-natal interventions centered on both the child and the mother has also been associated with better nutritional status. These include counseling on feeding and infant care practices (Bhandari et al. 2004; Shi et al. 2010), and the reduction of infectious diseases through interventions such as routine vaccination (Das et al. 2017; Rodriguez et al. 2011). The Ministry of Public Health of Ecuador have established guidelines for pre/post-natal care for all mothers and newborns (MSP 2016). These guidelines include iron prophylaxis in pregnant women and children, as well as systematic vaccination and counseling for pregnant women and mothers/fathers. The health system in Ecuador provides free iron prophylaxis for mothers and children who attend health services.

A greater number of episodes of diarrhea remained significantly associated with anemia after adjusting for the other variables including socioeconomic variables. Other studies have also demonstrated that diarrhea is a contributing factor to anemia among young children living in rural areas (Desai et al. 2005; Howard et al. 2007; Semba et al. 2008). This association can be explained by the effect of frequent episodes of diarrhea on the gastrointestinal tract. Moreover, it is well known that some etiological agents associated with diarrhea have an effect on the absorption of nutrients (Larsen et al. 2017).

With regard to chronic malnutrition, variables related to basic determinants such as family income presented a significant association which was independent of other variables. Children from families with a family income of less than $80 had a 2.74 times higher probability of having chronic malnutrition. This confirms the findings that show an inverse association between socio-economic situation and chronic malnutrition (Fink et al. 2017; Prendergast and Humphrey 2014; Tariku et al. 2017). On the other hand, maternal or paternal education, used as a *proxy* of socioeconomic level, was not associated with chronic malnutrition in our study as has been observed in other studies (Aheto et al. 2015; Black et al. 2013). Evidence suggests that it is the involvement of parents with the care of their children, beyond the parents’ educational level, that impacts their children’s growth (Kassebaum et al. 2017).

Maternal short stature was significantly associated with chronic malnutrition. Others studies confirm this finding and is explained by an intergenerational effect of malnutrition on the growth of children (Ferreira et al. 2008; Frojo et al. 2014; Lee et al. 2010; Ozaltin et al. 2010; Rahman and Chowdhury 2007; Ramakrishnan et al. 1999) and by genetic factors. In turn, this intergenerational effect is a product of conditions such as intrauterine growth retardation, poverty and other socio-cultural factors that determine feeding and care practices (Martorell and Zongrone 2012).

A greater number of children in the households were also significantly associated with chronic malnutrition. Mother’s total number of children influences the resources available for each child in financial terms, time, attention and exposure to infections (Sereebutra et al. 2006).

Excreta disposal in latrine, septic tank or outdoors, as well as river water consumption were significantly associated with a higher prevalence of chronic malnutrition and anemia (Aheto et al. 2015; Fikadu et al. 2014). This association was not independent of other socio-economic variables which show its indirect effect on chronic malnutrition and anemia through socio-economic status (Casale et al. 2018; Fikadu et al. 2014). Lack of access to clean water and effective sanitation are important causes of malnutrition (Fikadu et al. 2014; Fink et al. 2017; Prendergast and Humphrey 2014). These conditions directly affect health, innocuous food production and preparation and general hygienic practices.

One limitation of the study is the design, which limits the possibility of making cause-effect inferences. Some of the data included may have a memory bias, especially in areas related to feeding, care practices and presence of diarrhea, respiratory and parasitic infections. Children’s birth weights were not included in the study due to the difficulty of obtaining reliable data. However, this study included all the determinants from the causal model of malnutrition (UNICEF 1990) and its results are generalizable for children in San Isidro. Selection bias may be present due to selection of only participants enrolled in schools and day care centers.

This research suggests that comprehensive strategies that improve the socioeconomic situations of families are needed to reduce the prevalence of malnutrition and anemia. Empowering parents and strengthening maternal and child health services are necessary steps that will have a positive impact on the nutritional status of children under 5 years of age (Kassebaum et al. 2017; Muhoozi et al. 2018). To improve the nutritional status of the mother and the child is an unavoidable necessity to meet the Sustainable Development Goals (Larsen et al. 2017; WHO 2018).

## Conclusions

Socio-economic level, access to health services and previous biological characteristics like length at birth, were significantly associated with chronic malnutrition and anemia in children under five in San Isidro. Comprehensive strategies aimed at improving socioeconomic conditions, access to prenatal care, family planning and prevention from diarrheic diseases are needed in order to reduce the prevalence of chronic malnutrition and anemia in this rural population.
